# Flexible and Lightweight Carbon Nanotube Composite Filter for Particulate Matter Air Filtration

**DOI:** 10.3390/nano12224094

**Published:** 2022-11-21

**Authors:** Megha Chitranshi, Daniel Rui Chen, Peter Kosel, Marc Cahay, Mark Schulz

**Affiliations:** 1 Department of Electrical Engineering and Computer Science, University of Cincinnati, Cincinnati, OH 45221, USA; 2 Department of Mechanical and Materials Engineering, University of Cincinnati, Cincinnati, OH 45221, USA

**Keywords:** carbon nanotubes (CNTs), particulate matter (PM), air filtration

## Abstract

Particulate Matter (PM) has become an important source of air pollution. We proposed a flexible and lightweight carbon nanotube (CNT) composite air filter for PM removal. The developed CNT filtering layers were fabricated using a floating catalyst chemical vapor deposition (FC-CVD) synthesis process and then combined with conventional filter fabrics to make a composite air filter. Filtration performance for CNT filtering layer alone and composited with other conventional filter fabrics for particles size 0.3 μm to 2.5 μm was investigated in this study. The CNT composite filter is highly hydrophobic, making it suitable for humid environments. The CNT composite filter with two layers of tissue CNT performed best and achieved a filtration efficiency over 90% with a modest pressure drop of ~290 Pa for a particle size of 2.5 μm. This CNT composite filter was tested over multiple cycles to ensure its reusability. The developed filter is very light weight and flexible and can be incorporated into textiles for wearable applications or used as a room filter.

## 1. Introduction

Air pollution has become the biggest environmental health risk. According to the World Health Organization (WHO), air pollution kills an estimated seven million people every year. The data showed that 9 out of 10 people breathe air that exceeds guideline limits of high levels of pollutants [1]. EPA research on health effects from air pollution showed that air pollutants have detrimental effects on lungs and cause heart disease and other health problems [2]. There is a need to understand the adverse effects of air pollution and invent solutions to ensure healthy life and a sustainable environment. According to Fortune Business Insights, the global market for air filters stood at 12.10 USD billion in 2019 and is estimated to reach 20.63 USD billion by 2027 [3]. The increased demand and construction of green buildings is making air filtration an important part of a sustainable environment. In addition, the growth of the automobile industry will also increase the use of these filters in upcoming years.

Due to the large amount of particulate matter (PM) emission from the industrial sector, power plants, and other human activities, PM has become an important source of air pollution. PM consists of inorganic matter (sulfates, nitrates, etc.), organic matter (elemental carbon, organic carbon, etc.), in various sizes [4,5,6]. Based on particulate diameter, PM can be divided into PM 10 (aerodynamic diameter smaller than 10 µm) and PM 2.5 (aerodynamic diameter smaller than 5 µm) [7]. PM sized under 10 µm can pass through the body and enter the lungs, but PM sized under 2.5 µm are more harmful and can penetrate alveolus and blood vessels due to their small size [8]. Moreover, PM 2.5 can be present in the atmosphere for longer periods. To avoid the risk of exposure, capturing PM using filtration membrane is the commonly used solution [9,10,11,12,13]. In recent years, various CNT based particulate matter filters were studied due to their low-weight, high surface area and small pore size. Single walled carbon nanotubes (SWCNTs) of two different diameters were used to adsorb organic vapors [14]. It was observed that the amount adsorbed by tubes with narrow diameter is larger than the CNTs with wider diameter. The surface area of the narrow diameter tube was three times more than the wider diameter tubes. The higher adsorption amount was due to the enhanced interaction with adsorbate molecules in narrow diameter CNTs. The adsorption of volatile organic compounds (VOCs) on multi walled carbon nanotubes (MWCNTs) was also studied [15]. The presence of an amorphous carbon layer on the MWCNTs increased the adsorption property of the organic compounds on the MWCNTs. In passive capturing, filters block the pollutants with the help of dense filter structure and filter toxic pollutants by a colliding, attaching, and capturing mechanism [16]. A particulate air filter was fabricated by depositing CNT membranes on Si/SiO_2_ chips [17]. The permeability of the membranes was adjusted by the growth time. A filter with lower permeability showed 99% filtration efficiency for submicron size particulate. In another research, aligned CNT sheets were combined with polypropylene nonwoven fabric in a 3-layers cross-ply filter structure [18]. The developed filter was tested for particle diameters ranging from 0.01–0.3 μm at a face velocity of 10 cm/s. The filter showed a very high-quality factor and met the HEPA specifications. It was observed that by increasing the number of CNT layers, filtration efficiency increased along with an increase in pressure drop. A CNT-metal filter was fabricated by growing CNTs onto a conventional metal filter [19]. The filter with a bush-like CNTs nanostructure showed higher filtration efficiency.

In this paper, we demonstrated the use of a CNT network as a PM filter with very high efficiency. Different combinations of CNT layers and conventional filtering material were tested. The stability of the filter was investigated for multiple filtration cycles. This flexible, high efficiency filter can be incorporated into textiles or as a room filter.

## 2. Materials and Methods

### 2.1. Synthesis of Thin CNT Filtering Layer

The thin CNT sheet was synthesized using the floating catalyst chemical vapor deposition (FC-CVD) method. The FC-CVD method is a one-step method and produces large-scale nanotube material. The fuel used in the synthesis process consists of a carbon precursor and catalyst. Sulfur was used as a promoter and injected into a tube in a high-temperature furnace (1400 °C). The fuel is a mixture of methanol (Thermo Fisher Scientific, MA, USA), thiophene (Sigma-Aldrich, Inc., St. Louis, MO, USA), and ferrocene (Sigma-Aldrich, Inc., St. Louis, MO, USA). The fuel injection rate varied from 30 mL/hr to 60 mL/hr. The fuel injection was carried out with the help of an atomizer which injected a fine mist of fuel at the inlet of the reactor. The power of the atomizer is self-driven and varies according to the applied load. Hydrogen and argon gases were used as carrier gases, the flow for hydrogen was 100 sccm, and for argon it varied from 500–2000 sccm. The CNT sheet was collected on the other end of the tube on a rotating drum to form a thin sheet. The thickness of the sheet can be controlled by varying the collection time. Other details about the synthesis process can be found in previous publications [20,21,22,23,24,25,26,27,28,29,30].

### 2.2. Filter Fabrication

The filter was prepared by collecting a very thin layer of CNT sock on the base carbon fiber tissue layer and melt blown fabric. CNT filters are easily clogged by the PM due to the very small space between the CNTs. Using a porous material as a base layer provides a microporous structure with structural stability while CNT increases the surface area of the filter [11]. Single layers and various composites were tested for better filtration efficiency.

### 2.3. PM Generation and Measurement

In this study, we have used incense smoke as a PM source. These PM were captured using a CNT composite filter. The experiments were conducted for 20 min over several days and months. After the filtration experiment, the tested layers were examined with SEM and EDX for a before and after comparative analysis. A particle counter was also used at the outlet of the filter to record the concentration of the PM particles.

### 2.4. Filter Efficiency Measurement

The performance of the air filter was evaluated using two key factors: filtration efficiency and pressure-drop. To calculate the efficiency of the filter, input and output concentration of the PM were measured using the particle counter.

The filtration efficiency was calculated using the below equation:

(1) $$\mathrm{Filtration}\;\mathrm{Efficiency}=1-\frac{C_{out}}{C_{in}}$$

where *C_in_* is the measured PM concentration without a filter and *C_out_* is the maximum PM concentration measured after the filtration.

The Quality Factor was calculated using the following equation:

(2) $$\mathrm{Quality}\;\mathrm{Factor}\;\left(Q_{f}\right)=\frac{-\ln\;\left(1-E\right)}{\Delta P}$$

where *E* is the filtration efficiency and Δ*P* is the pressure drop.

## 3. Results and Discussion

**Figure 1:**
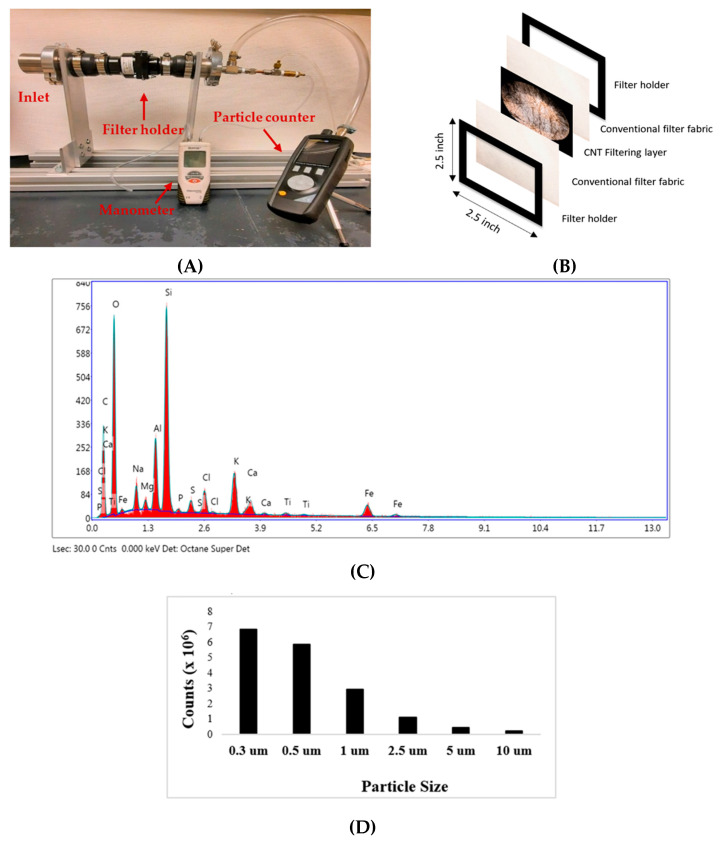
(**A**) The experimental setup, (**B**) a schematic diagram of CNT composite filter structure, (**C**) EDX spectrum of PM, and (**D**) PM concentration.

The experimental setup for PM filtration is shown in Figure 1A. PM smoke was introduced at the inlet of the tube. The CNT composite filter was placed at the center. A particle counter to record the PM concentration was attached to the outlet of the tube. To reduce the back flow at the inlet, suction was applied at the outlet side. Before starting the experiment, PM particles using incense smoke filled the inlet and then continuously introduced PM particles during the experiment. An inlet view with a CNT filter inside is shown in Figure 1B. The size of the holder is 2.5 × 2.5-inch.

The surface morphology and composition of the particles from incense smoke is shown in Figure 1C. An EDX analysis was performed on different spots of the sample. The EDX analysis showed that the smoke mainly consisted of O, Si, Al, Na, Ca, K, Mg, and other elements. It is clear from Figure 1D that the majority of the PM particles from the incense smoke are small particles with diameter smaller than 2.5 μm. A particle counter was used to measure the concentration of the PM from incense smoke.

### 3.1. Single-Layer Performance Analysis

**Figure 2:**
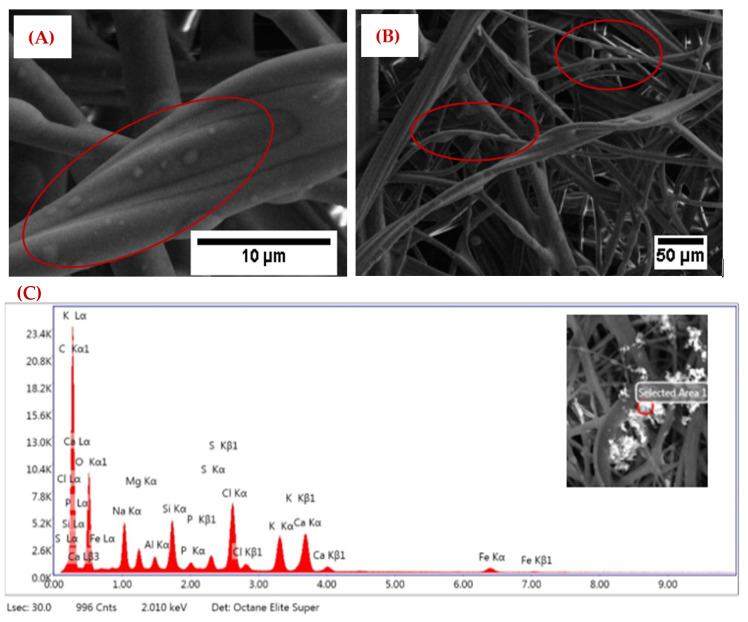
The melt blown filtering layer (**A**) An SEM image of captured PM at 10 μm, (**B**) at 50 μm, and (**C**) the EDX spectrum of captured PM.

Three different single layers were tested: melt blown (Single layer of melt blown conventional filtering fabric), thin CNT on thick tissue (thin layer of CNT was collected on the base carbon fiber tissue layer), and thin CNT with Granulated Activated Carbon (GAC) (thin layer of CNT with GAC nanoparticles incorporated during the synthesis process). The filter captured the PM with a combination of different mechanisms such as Brownian diffusion, interception, inertia impact, and electrostatic deposition [11,16,31]. SEM and EDX analyses were performed on the tested layer to determine the morphology of the captured PM particles. The captured PM particles on the melt blown single layer is shown in Figure 2. The color of the melt blown layer was changed after the filtration experiment. The SEM images of captured PM particles from incense smoke are shown in Figure 2A,B, for 10 μm and 50 μm size range. The EDX analysis (Figure 2C) showed the peaks for Na, Mg, Ca, Al, Si, S, Cl, P, and other PM in our tested sample.

**Figure 3:**
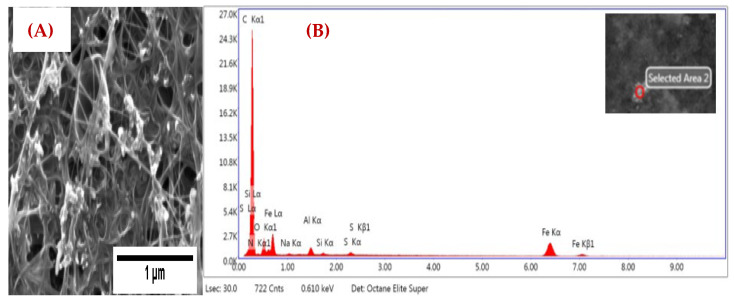
CNT on a thick tissue filtering layer (**A**) SEM image of captured PM at 1 μm, and (**B**) EDX spectrum of captured PM.

The captured PM particles on thin CNT for a thick tissue sample are shown in Figure 3. The SEM images of captured PM particles from incense smoke of 1 μm size is shown in Figure 3A. From the EDX analysis (Figure 3B), it is clear that the sample trapped particular matter from the incense smoke. Peaks for Na, Al, Si, S, O, and K particulate matter in our tested sample are clearly visible.

**Figure 4:**
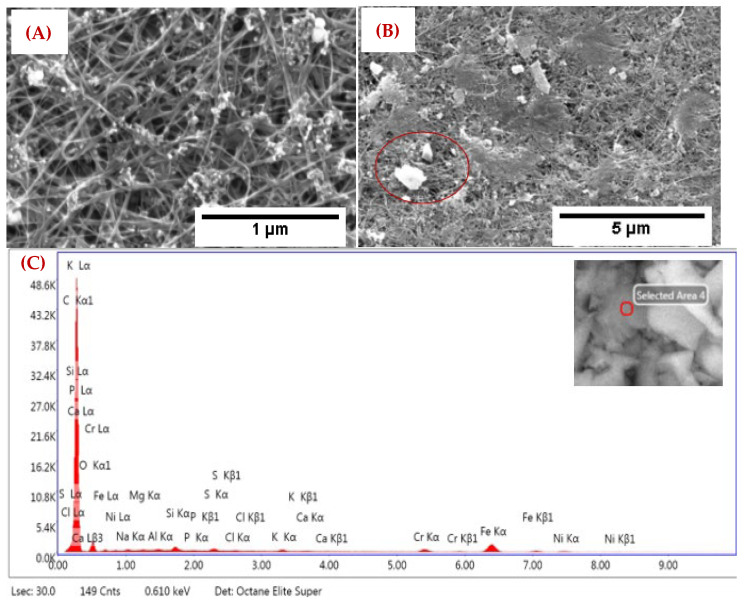
The CNT-GAC filtering layer; (**A**) an SEM image of captured PM at 1 μm, (**B**) at 5 μm, and (**C**) the EDX spectrum of captured PM.

The captured PM particles on thin CNT-GAC on a thick tissue sample are shown in Figure 4. The SEM images of captured PM particles from incense smoke on the top layer and middle part are shown in Figure 4A,B. An EDX analysis was performed on different spots of tested sample layers. From the EDX analysis (Figure 4C), we can see the peaks for Na, Mg, Al, Si, S, Cl, P, Ca, Cr, and other particulate matter in our tested sample. The CNT-GAC sample trapped more particles compared to other tested single layer filters.

**Figure 5:**
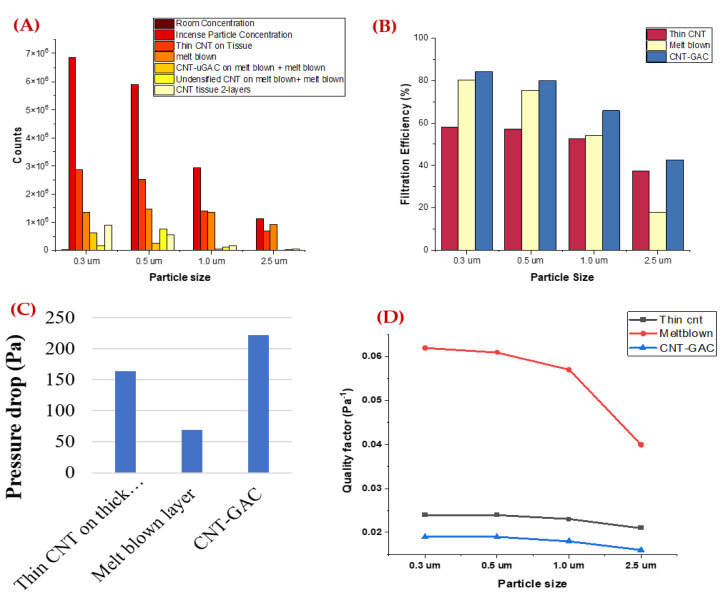
Single-layer filter performance (**A**) PM concentration, (**B**) Filtration Efficiency, and (**C**) Pressure drop of filters, and (**D**) Quality Factor.

From Figure 5A, the concentration of the PM particles from incense smoke are greater for a particle size smaller than 2.5 μm. Single layers of three samples were tested and the output concentration of PM particles were recorded using a particle counter. The filtration efficiency of these three samples for a particle size of 0.3 μm to 2.5 μm is shown in Figure 5B. A sample with thin CNT on thick tissue performed poorly and had less than 60% filtration efficiency for all particle sizes. For sizes of 1 μm and 2.5 μm, the filtration efficiency from this sample was lower than 60%. The single layer of melt blown performed at 80% filtration efficiency for a particle size of 0.3 μm. For sizes 1 μm and 2.5 μm, the filtration efficiency of melt blown was lower than 60%. The sample with CNT-GAC performed best among all the three samples with filtration efficiency greater than 80% for a particle size of 0.3 μm and 0.5 μm. Figure 5C shows the pressure drop of the filter samples. 

The CNT-GAC filtering layer showed a very high pressure drop. The pressure drops of the filters were significantly increased with continuous loading of the PM during the experiment. The quality factor provides the overall performance of the filtering material by including both pressure drop and filtration efficiency parameters. The higher the quality factor, the better the performance of the filtering material [32]. In Figure 5D, melt blown material has a very high-quality factor compared to other filtering materials.

It is clear that single layers are not sufficient to filter all the particulate matter from smoke. The as-synthesized CNT sheet is not uniformly aligned, which creates gaps between the nanotube strands. The PM can easily pass through these gaps [33]. Therefore, multiple layers in different combinations (CNT with melt blown or other commercially available material) are required to fill these gaps and improve the filtration efficiency.

### 3.2. Composite Filter Performance Analysis

**Figure 6:**
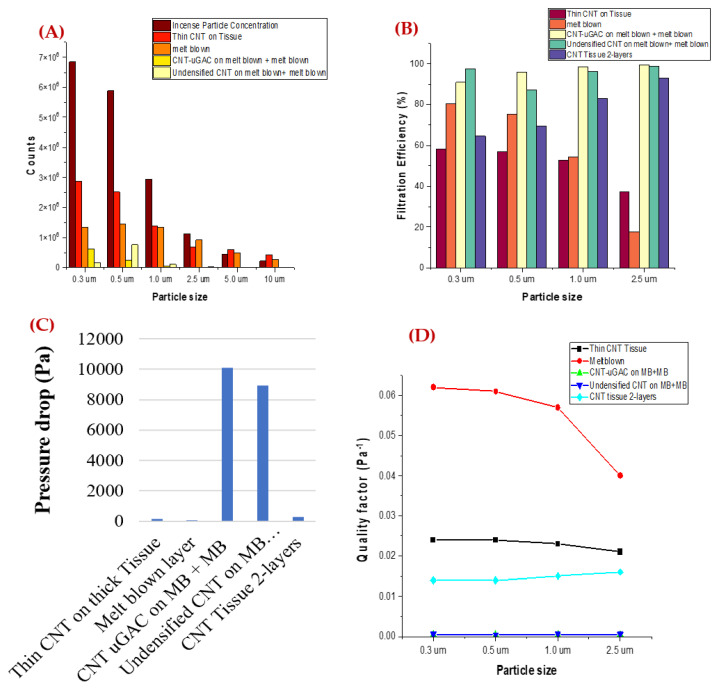
Composite filter performance (**A**) PM concentration, (**B**) Filtration Efficiency, and (**C**) Pressure drop of filters, and (**D**) Quality Factor.

For a composite filter, samples with 2-layers of thin CNT on carbon fiber tissue, undensified CNT on melt blown with melt blown outer layer, and CNT with micro-granulated activated carbon (CNT-GAC) with an outer layer of melt blown were tested. Undensified CNT is a CNT sheet without any alcohol densification. During the CNT sheet synthesis process, we use acetone for densification while collecting our CNT. The densification process makes CNT collection easier but also reduces the surface area. We investigated the filtration performance of both types of CNT sheets: with densification (normal as-synthesized CNT sheet) and without densification (Undensified CNT). The concentration of the PM is higher for a particle size range from 0.3 μm to 1 μm (Figure 6A). The sample with CNT-GAC showed very high filtration efficiency, but the pressure drop of the sample was also very high. Among all the samples (Figure 6B,C), the CNT-Tissue with two layers showed good performance with a moderate pressure drop of ~286 Pa. The filtration efficiency of the filter for a particle size of 2.5 μm reached up to 93%. For this study, we concluded that CNT-Tissue with two layers is the best composite filter for PM air filtration for a particle size of 2.5 μm. The low pressure drop of this composite filter may be due to its two-layer structure, which provided enough separation between the CNT layers for efficient air flow. By adding the second CNT-Tissue layer, the thickness of the filter increased, which also improved the filtration efficiency [34]. Figure 6D shows the quality factor of the filters. There is a need to understand the filtration mechanism for a particle size below 2.5 μm for our filter and try to improve the overall performance of the filter.

**Figure 7:**
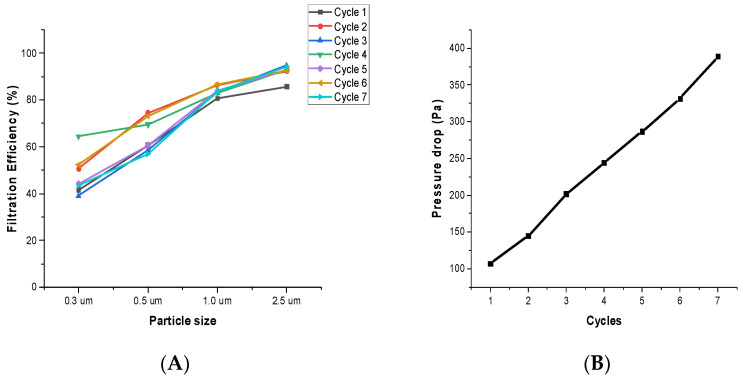
(**A**) Filtration Efficiency, and (**B**) pressure drop of the filter for seven cycles.

To ensure the stability of the composite filter, the performance of the CNT-Tissue with two layers was investigated over multiple cycles (Figure 7). The filtration performance (Figure 7A) did not vary much, but the pressure drop (Figure 7B) of the filter increased continuously with continuous loading. The increase in pressure drop of the filter due to the clogging of the filter with continuous loading is also supported by Thomas et.al’s study [35]. This filter can be useful in different applications. Firefighters often work in harsh environments and encounter hazardous pollutants. To reduce the risk of exposure, this CNT filter can be incorporated into firefighter uniforms to prevent any toxic particles from entering the fabric. Several methods for integrating the CNT sheet with available conventional textiles have been studied in our previous research [22,26]. The safety of the CNT sheet is discussed in our previous publication [24], in which it is advised that a veil or outer layer can be used with CNT sheets for safety purposes.

## 4. Conclusions

In this paper, a composite filter was fabricated using the randomly distributed CNTs in a nonwoven sheet with other conventionally available filtering materials. To provide better air flow, a thin layer of CNT was collected on a very thin carbon fiber tissue layer, using it as a base layer. The filtration efficiency for different combinations of CNT layers alone and composited with commercially available filter fabric were investigated for a particle size varying from 0.3 μm to 2.5 μm. The developed CNT composite filter with two layers of Tissue CNT performed best for a particle size of 2.5 μm and achieved a filtration efficiency of over 90% with a moderate pressure drop of ~290 Pa. The filtration efficiency of the filter did not change much while using it for a longer period. Apart from air purification, it can possibly be integrated in textiles due to its light weight and flexibility for wearable applications.

## Data Availability

The data that support the findings of this study are available from the corresponding author upon reasonable request.
